# Prevalence and trends of cancer-related daily life limitations among gastrointestinal cancer survivors

**DOI:** 10.1007/s11764-025-01833-1

**Published:** 2025-05-24

**Authors:** Azza Sarfraz, Abdullah Altaf, Mujtaba Khalil, Zayed Rashid, Shahzaib Zindani, Areesh Mevawalla, Timothy M. Pawlik

**Affiliations:** Department of Surgery, The Ohio State University Wexner Medical Center and James Comprehensive Cancer Center, Columbus, OH USA

**Keywords:** Gastrointestinal cancer, Survivorship, Functional limitations, Activity limitations, Health disparities

## Abstract

**Purpose:**

The number of gastrointestinal (GI) cancer survivors has increased substantially due to improvements in early detection and treatment, yet long-term functional patient outcomes remain poorly characterized. We sought to quantify the burden of activity limitations (AL) and functional limitations (FL) among GI cancer survivors compared with non-GI cancer survivors and the general U.S. population, as well as identify key predictors of cancer-related limitations.

**Methods:**

The National Health Interview Survey (NHIS), a nationally representative dataset (1997–2023), was queried to examine the prevalence and trends of cancer-related limitations among GI cancer survivors, non-GI cancer survivors, and the general U.S. population. Multivariable logistic regression analyses identified independent predictors of AL and FL, adjusting for demographic and socioeconomic variables.

**Results:**

Among 5,513 GI cancer and 39,887 non-GI cancer survivors, 50.2% (Relative Risk [RR]: 1.23, 95% CI: 1.19–1.27) and 70.7% (RR: 1.07, 95% CI: 1.05–1.09) of GI cancer survivors reported AL and FL, respectively, compared with non-GI cancer survivors. The general U.S. population had a markedly lower prevalence of AL (13.5%; RR: 0.33, 95% CI: 0.33–0.34) and FL (35.7%; RR: 0.54, 95% CI: 0.53–0.54). GI cancer survivors were older (mean age: 69.1 vs. 65.3 vs. 36.1 years), more often single (8.8% vs. 8.6% vs. 28.3%), and more frequently received public insurance (75.1% vs. 68.9% vs. 27.4%) compared with non-GI cancer survivors and the general population (*p* < 0.05). In multivariable analysis, GI cancer survivors had 21% higher odds of AL (OR: 1.21, 95% CI: 1.11–1.32, *p* < 0.001) and 11% higher odds of FL (OR: 1.11, 95% CI: 1.00–1.19, *p* = 0.049).

**Conclusions:**

GI cancer survivors face a significantly higher burden of functional limitations, influenced by demographic and socioeconomic factors.

**Implications for Cancer Survivors:**

Addressing functional disparities through targeted rehabilitation and support services may improve long-term outcomes.

**Supplementary Information:**

The online version contains supplementary material available at https://doi.org/10.1007/s11764-025-01833-1.

## Introduction

Gastrointestinal (GI) cancers —defined as individuals who have been diagnosed with and treated for malignancies of the esophagus, stomach, liver, gallbladder, pancreas, colon, or rectum—represent a major and growing patient population in the United States (US), with an increasing incidence over the past several decades [1]. GI cancers can impact patient quality of life (QoL), particularly among individuals with advanced disease and/or patients undergoing multimodal therapy [2, 3]. Treatment-related complications, including cachexia, chronic pain, nutritional deficiencies, and fatigue, frequently contribute to persistent functional impairments [4, 5]. Surgical procedures such as colectomy, gastrectomy, and pancreatectomy may necessitate long-term adaptations, including ostomies or enteral feeding, that further affect daily living and independence [6, 7]. Even among patients with early-stage disease, curative treatments can result in lasting physical deficits that limit mobility, social engagement, and overall well-being [8]. As a result, GI cancer survivors may struggle to maintain their pre-diagnosis level of functionality [9, 10].

Although survival remains the dominant metric in oncology, mortality outcomes alone fail to capture the broader quality-of-life challenges faced by survivors [11]. Many patients—particularly older adults—prioritize maintaining functional independence and daily activity over survival alone [12, 13]. Functional disabilities often arise as a consequence of treatment-related complications and can have a lasting impact on QoL and independence of survivors [14]. To date, the long-term functional consequences of GI cancer remain poorly characterized [15]. Furthermore, complex factors such as age, sex, and marital status may interact to influence recovery trajectories, but these dynamics have received limited attention [16, 17]. National cancer-related healthcare expenditures have been estimated to be $158 billion in 2020, a burden expected to rise with improving survival and prolonged recovery needs [18]. However, the economic burden is not distributed equally. Prior studies have demonstrated that insurance type, income level, employment status, citizenship, and race/ethnicity influence access to care, receipt of rehabilitative services, and long-term survivorship outcomes [19–21]. Immigrant populations and racial/ethnic minorities often face additional barriers, resulting in higher risk of disability and unmet survivorship needs [22–24]. A deeper understanding of these disparities is essential to inform equitable survivorship care and minimize long-term functional impairments [25].

Therefore, we sought to characterize the prevalence and trends of self-reported cancer-related activity and functional limitations among GI cancer survivors compared with non-GI cancer survivors and the general US population. In addition, using nationally representative data from the National Health Interview Survey (NHIS), we sought to identify factors influencing the likelihood of cancer-associated functional impairments among GI cancer survivors.

## Methods

### Data source and study population

The NHIS is a nationally representative, cross-sectional survey administered annually by the Centers for Disease Control and Prevention (CDC) to assess population health trends in the US [26]. The NHIS employs a complex, multi-stage clustered sampling design to ensure geographic coverage and representativeness. Data collection is conducted through structured in-person interviews with a randomly selected adult from each participating household. The NHIS dataset was accessed through the Integrated Public Use Microdata Series (IPUMS), a service provided by the University of Minnesota that harmonizes publicly available survey data [27]. The most recent NHIS data from 1997 to 2023 were utilized to investigate the prevalence of cancer-related limitations among cancer survivors. The study population included adults (≥ 18 years) who self-reported a cancer diagnosis in the NHIS Sample Adult survey. Individuals diagnosed with non-melanoma skin cancer, blood cancers (leukemia, lymphoma, myeloma), or brain cancer were excluded from the analysis. Blood cancers (leukemia, lymphoma, myeloma) and brain cancers were excluded due to their distinct disease courses, treatment regimens, and mechanisms of functional impairment, which differ from solid tumors and may not be directly comparable. The study was deemed exempt from institutional review board (IRB) approval as all data were publicly available and de-identified. The methodology adhered to the Strengthening the Reporting of Observational Studies in Epidemiology (STROBE) guidelines.

### Covariates and outcomes of interest

Data on individual demographics including age, sex, race, ethnicity, citizenship, state of residence, and year of survey were extracted. For race, individuals self-identified as White, Black, or other; for ethnicity, individuals were categorized as Hispanic or non-Hispanic; for citizenship, individuals were defined as US Citizen or Immigrant. Income was categorized into three groups including below the poverty line, low to middle income, and high income; data on the number of hours worked per week were also obtained. Insurance type was classified into Medicare, Medicaid, other public, private, and uninsured. Other public insurances include all non-Medicaid and non-Medicare government-sponsored health insurance recorded by NHIS, including Indian Health Services and military health insurance plans. Participants were classified based on their marital status as married, single, divorced/separated, or widowed.

The primary exposure of interest was a diagnosis of GI cancer (Supplementary Table 1). GI cancers included esophageal, stomach, gallbladder, liver, pancreatic, colon, and rectal cancers. Participants were asked if they had any limitations due to a chronic health condition (yes/no), with limitations defined as any difficulty in performing a normal activity without special equipment or assistance. Individuals who responded"yes"were further asked to indicate which chronic health condition/s were the cause of their limitation, including cancer. The primary outcome of interest was cancer-related limitations, which were determined using the “LANY” (any activity limitation [AL]) and “FLANY” (any functional limitation [FL]) variable. AL and FL were captured using separate NHIS questions and analyzed as two distinct outcomes. Data regarding respondents'limitations were extracted only for individuals who self-reported a cancer diagnosis. AL were defined as self-reported difficulty to perform basic daily activities due to a physical, mental, or emotional problem; FL were defined as self-reported difficulty performing broader physical, cognitive, or social tasks essential for independent living, as previously reported [28].

Data on overall disability status were also extracted using the Washington Group Short Set Composite Disability Indicator, which was determined by the “DISABWGSS” variable [29]. This measure identifies individuals at a greater risk than the general population for experiencing restrictions in participation due to difficulties performing universal, basic actions. Respondents were classified as having a disability if they reported"a lot of difficulty"or being"unable to do"any of the following six activities: vision, hearing, walking or climbing steps, communicating in their usual language, washing or dressing, or remembering or concentrating. Participants were asked whether they had limitations in performing activities of daily living (ADLs) using the “LADL” variable, which include dressing, bathing, toileting, transferring, and eating, as well as instrumental activities of daily living (IADLs), such as shopping, housework, managing finances, and using transportation, using the “LAIADL” variable. These variables were included as additional descriptive measures of disability but were not incorporated into the primary outcomes of AL or FL. Additionally, participants self-reported limitations in socializing (FLSOCIAL), relaxing or engaging in leisure activities (FLRELAX), walking without special equipment (LAWALK), climbing stairs (FLCLIMB), standing for prolonged periods (FLSTAND2HR), and sitting for extended durations (FLSIT2HR).

### Statistical analyses

Descriptive statistics for categorical variables were presented as frequencies and percentages (%) and were analyzed using the Chi-square test or Fisher's exact test, as appropriate. Continuous variables were reported as median values with interquartile ranges (IQR); comparisons between groups were conducted using the Kruskal–Wallis test. Unadjusted rates of AL and FL were calculated for GI cancer survivors, non-GI cancer survivors, and the general US population. Using non-GI cancer survivors as the reference group, unadjusted relative risks (RR) with 95% confidence intervals (CI) were estimated for the presence of AL and FL. Multivariable logistic regression analysis was performed to identify factors independently associated with AL and FL among cancer survivors. The regression models were adjusted for age, sex, race, ethnicity, citizenship status, income level, insurance status, marital status, employment, and hours worked per week to account for potential confounders. Subgroup analyses were conducted to examine whether the association between cancer type and cancer-related limitations varied by sex, age group, and socioeconomic status. Sampling weights provided by NHIS were applied to all regression models to produce population-representative estimates. All statistical tests were two-tailed, and the significance level was set at *p* ≤ 0.05. All statistical analyses were performed using Stata version 18.0 (StataCorp, College Station, TX).

## Results

### Baseline characteristics

Among 2,295,201 survey respondents in the unweighted sample (weighted sample: 141,344,142 persons), 5,513 (0.2%) were GI cancer survivors and 39,887 (1.6%) were non-GI cancer survivors. GI cancer survivors were older than both non-GI cancer survivors and the general US population (mean age: GI cancers survivors: 69.1 years (± 12.9) vs. non-GI cancer survivors: 65.3 years (± 14.9) vs. general US population: 36.1 years (± 22.5); *p* < 0.001). The proportion of male participants was similar between GI cancer survivors (48.9%) and the general US population (49.2%), while a lower proportion of non-GI cancer survivors (34.5%) were men (*p* < 0.001). A vast majority of GI cancer survivors (85.7%) and non-GI cancer survivors (87.2%) identified as White compared with a lower proportion in the general US population (78.9%) (*p* < 0.001). Overall, 43.2% of GI cancer survivors reported being married versus 46.1% of non-GI cancer survivors and 56.2% of the general US population (*p* < 0.001). GI cancer survivors were more likely to have Medicare coverage (70.5%) compared with non-GI cancer survivors (60.8%), while non-GI cancer survivors were more likely to have Medicare versus the general U.S. population (13.5%) (*p* < 0.001). Of note, GI cancer survivors worked fewer hours per week (mean, 8.9 ± 18.5) compared with non-GI cancer survivors (mean, 11.8 ± 19.8) and the general US population (mean, 19.1 ± 23.2) (*p* < 0.001) (Table 1).

**Table 1:** Baseline characteristics of GI cancer survivors, non-GI cancer survivors and US general population

Characteristics	US Population (*n* = 2,204,819, 98.2%)	GI Cancer Survivors (*n* = 5,513, 0.2%)	Non-GI Cancer Survivors (*n* = 39,887, 1.6%)
Sex (male)	1,069,318 (49.2)	2,727 (48.9)	13,058 (34.5)
Age (years), mean	36.1 (22.5)	69.1 (12.9)	65.3 (14.9)
< 50	1,545,658 (70.1)	448 (10.6)	6,208 (17.4)
≥ 50	659,161 (29.9)	5,065 (89.4)	33,661 (82.6)
BMI (kg/m^2^), mean	27.1 (5.3)	27.4 (5.4)	27.5 (5.4)
< 18.5	13,914 (1.9)	194 (2.9)	870 (2.2)
18.5–24.9	273,412 (36.2)	1,813 (33.1)	12,897 (33.1)
25–29.9	261,054 (34.5)	1,876 (35.4)	13,452 (35.2)
≥ 30	205,551 (27.4)	1,490 (28.6)	11,406 (29.5)
Race			
White	1,513,870 (78.9)	4,180 (85.7)	31,540 (87.2)
Black	279,933 (9.7)	623 (9.7)	3,882 (8.8)
Other	200,759 (8.4)	285 (4.6)	1,756 (4.0)
Ethnicity			
Non-Hispanic	1,717,972 (83.7)	5,074 (92.7)	36,991 (93.6)
Hispanic	485,782 (16.3)	438 (7.3)	2,862 (6.4)
Citizenship			
Citizen	1,903,298 (91.4)	5,154 (98.1)	37,484 (98.4)
Immigrant	179,832 (8.6)	118 (1.9)	771 (1.6)
Marital status			
Married	898,810 (56.2)	2,302 (43.2)	17,796 (46.1)
Single	450,427 (28.3)	478 (8.8)	3,329 (8.6)
Divorced/separated	172,544 (9.9)	954 (16.7)	7,884 (20)
Widowed	95,658 (5.6)	1,585 (31.3)	9,243 (25.3)
Income			
Below poverty line	280,708 (13.6)	709 (14.4)	4,565 (12.9)
Low to middle income	377,278 (18.7)	1,171 (25.0)	7,527 (21.1)
High income	1,199,063 (67.7)	2,820 (60.6)	23,216 (66.0)
Insurance type			
Medicare	295,954 (13.5)	3880 (70.5)	24,272 (60.9)
Medicaid	263,570 (10.7)	266 (4.6)	2,011 (5.0)
Other public	68,271 (3.2)	41 (0.9)	467 (1.2)
Private	1,176,038 (58.6)	1,026 (20.1)	10,404 (26.1)
Uninsured	325,152 (14.0)	189 (3.9)	1,993 (5.0)
Hours worked per week	19.1 (23.2)	8.9 (18.5)	11.8 (19.8)

*All *p*−values are <0.001. N represents the unweighted number of survey respondents. Weighted percentages are reported

*BMI *body mass index, *GI *gastrointestinal, *HS *high school, *kg/m2 *kilograms per square meter, *US *United States

### Cancer-associated limitations among GI cancer survivors

**Fig. 1:**
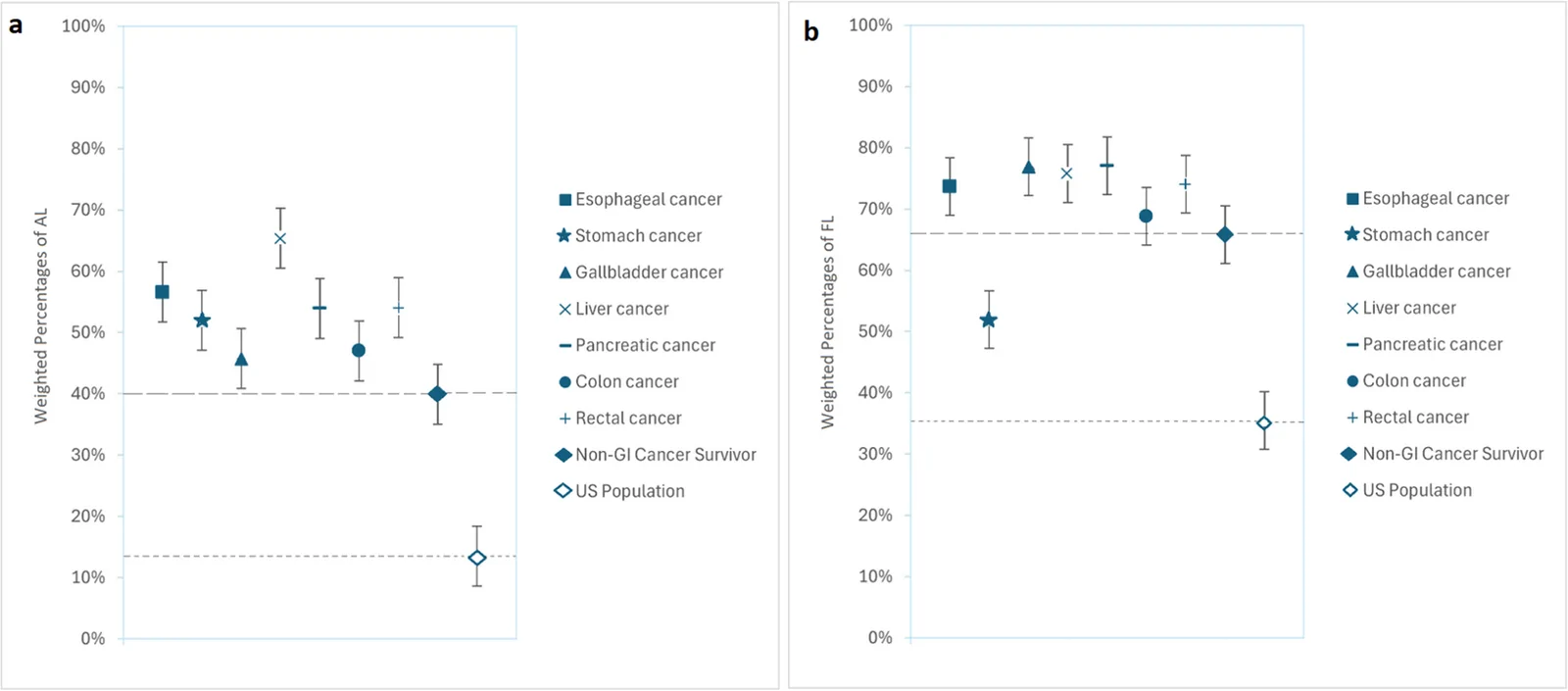
Prevalence of **a**) activity limitations (AL) and **b**) functional limitations (FL) among gastrointestinal (GI) cancer survivors by cancer site compared to non-GI cancer survivors and the general U.S. population. Weighted percentages and standard errors shown

Overall, 50.2% of GI cancer survivors reported at least one AL versus 40.8% of non-GI cancer survivors and 13.5% of the general US population (*p* < 0.001) (Table 2). On unadjusted analysis, GI cancer survivors had 23% higher RR of experiencing any AL compared with non-GI cancer survivors, while the general US population had a 67% lower RR (*p* < 0.001). Figure 1a illustrates the variation in AL prevalence across GI cancer types, with liver, esophageal, and pancreatic cancer survivors having the highest prevalence. While colon cancer survivors had the lowest AL among GI cancer survivors, the prevalence was still higher than AL rates among non-GI cancer survivors and the general US population. Moreover, any self-reported FL was also more prevalent among GI cancer survivors (70.7%) compared with non-GI cancer survivors (66.2%) and the general US population (35.7%) (*p* < 0.001). Relative to non-GI cancer survivors, the unadjusted risk of any FL was 7% higher among GI cancer survivors and 46% lower among the general US population (*p* < 0.001). Figure 1b illustrates the variation in FL prevalence among GI cancer types, with liver and pancreatic cancer survivors experiencing the highest rates.

**Table 2:** Comparison of prevalence and relative risk of activity limitations (AL) and functional limitations (FL) among GI cancer survivors, non-GI cancer survivors and US general population

Variables	US Population (*n* = 2,250,201)	GI Cancer Survivors (*n* = 5,513)	Non-GI Cancer Survivors (*n* = 39,887)
Any AL	250,211 (13.5)	2,092 (50.2)	11,858 (40.8)
Unadjusted relative risk of AL	0.33 (0.33–0.34)	1.23 (1.19–1.27)	Reference
Any FL	218,474 (35.7)	3,028 (70.7)	19,834 (66.2)
Unadjusted relative risk of FL	0.54 (0.53–0.54)	1.07 (1.05–1.09)	Reference
Need help with ADLs	31,706 (1.6)	373 (8.5)	1,779 (5.8)
Need help with IADLs	55,582 (3.7)	741 (17.0)	3,839 (12.6)
Unable to work	103,668 (7.0)	1,143 (26.2)	5,880 (19.2)
Difficulty remembering	45,169 (2.2)	450 (10.3)	2,543 (8.3)
Difficulty socializing	48,919 (7.9)	968 (24.0)	5,501 (19.1)
Difficulty relaxing	26,405 (4.2)	482 (11.1)	2,711 (9.0)
Difficulty walking	74,635 (3.7)	963 (22.0)	5,263 (17.2)
Difficulty climbing stairs	93,534 (14.6)	1,843 (41.1)	11,214 (35.6)
Difficulty maintaining standing	125,111 (20.0)	2,116 (51.3)	13,046 (44.8)
Difficulty maintaining sitting	72,114 (11.4)	980 (22.8)	6,526 (21.6)
Washington Composite Disability Indicator	13,404 (9.6)	261 (22.9)	1,836 (19.7)

*All *p*−values are <0.001

According to the Washington Composite Disability Indicator, disability was most prevalent among GI cancer survivors (22.9%) followed by non-GI cancer survivors (19.7%) and the general US population (9.6%) (*p* < 0.001). Of note, Supplementary Fig. 1 depicts the most common cancer-related limitations reported by GI cancer survivors with standing (51.3%), climbing stairs (41.1%), and socializing (24.0%) being the most frequently reported impairments. Difficulties with working (26.2%), sitting (22.8%), and walking (22.0%) were also prevalent, while being unable to perform ADLs (8.5%) was the least commonly reported limitation but remained higher than observed in non-GI cancer survivors and the general US population.

**Fig. 2:**
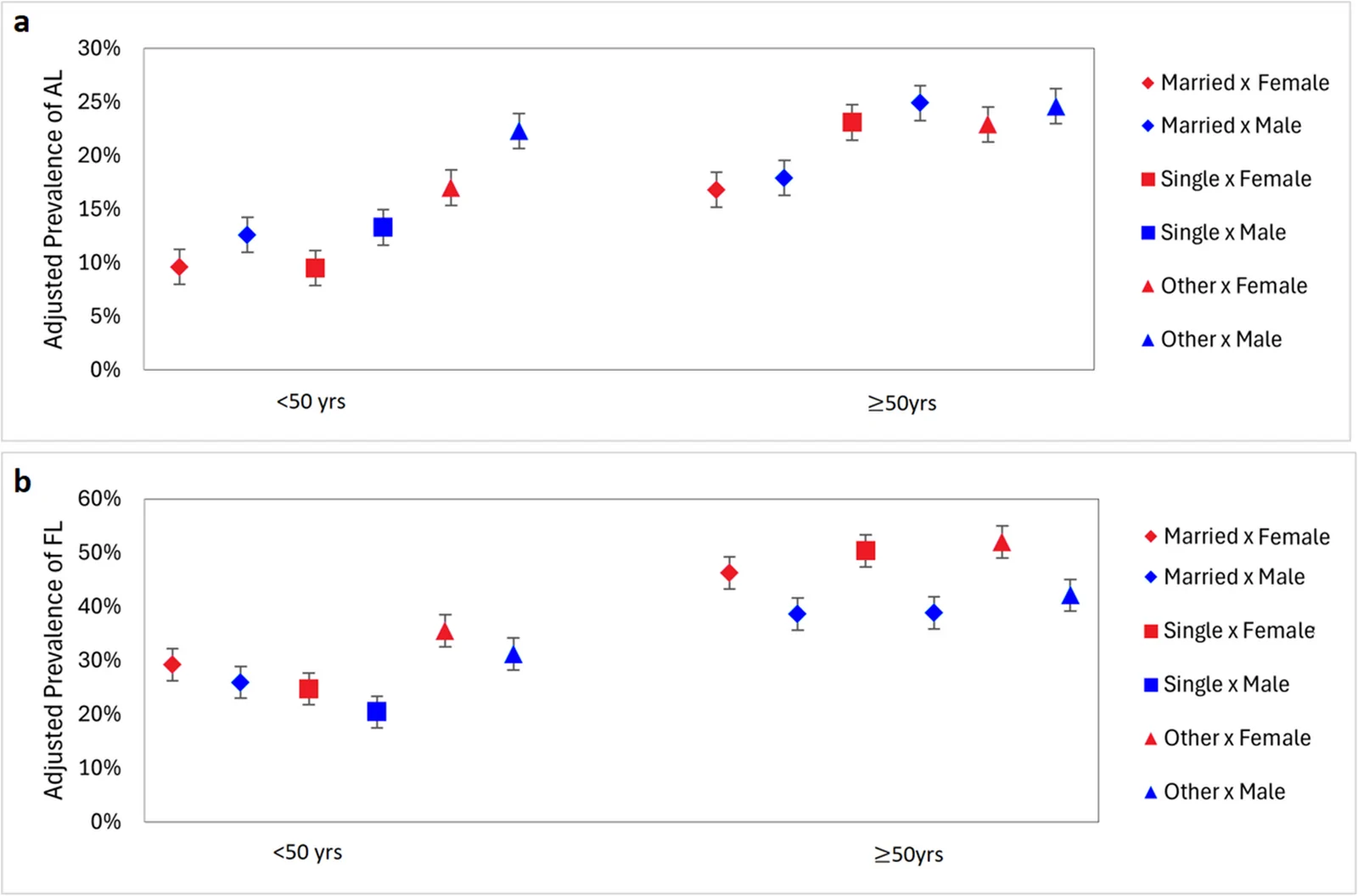
Adjusted prevalence of **a**) activity limitations (AL) and **b**) functional limitations (FL) among gastrointestinal (GI) cancer survivors, stratified by sex, marital status, and age. *Other: Divorced/Separated/Widowed

### Factors associated with cancer-related limitations

After adjusting for competing risk factors, GI cancer survivors had 21% higher odds of AL than non-GI cancer survivors (OR: 1.21 [95% CI: 1.11–1.32], *p* < 0.001) (Supplementary Table 2). Older age (≥ 50 years, OR: 1.36 [95% CI: 1.23–1.52], *p* < 0.001), Medicaid coverage (OR: 1.51 [95% CI: 1.30–1.74], *p* < 0.001), and being divorced/separated (OR: 1.64 [95% CI: 1.52–1.77]) were independently associated with increased odds of AL. In contrast, female sex (OR: 0.88 [95% CI: 0.82–0.93], *p* < 0.001), Hispanic ethnicity (OR: 0.86 [95% CI: 0.77–0.96], *p* = 0.009), high income (OR: 0.37 [95% CI: 0.33–0.40], *p* < 0.001), private insurance (OR: 0.76 [95% CI: 0.69–0.83], *p* < 0.001), and full-time employment (OR: 0.22 [95% CI: 0.20–0.25], *p* < 0.001) were protective factors.

Similarly, after adjusting for covariates, GI cancer survivors had 11% higher odds of FL than non-GI cancer survivors (OR: 1.11 [95% CI: 1.00–1.19], *p* = 0.049) (Supplementary Table 3). Female sex (OR: 1.40 [95% CI: 1.31–1.49]), older age (≥ 50 years, OR: 1.69 [95% CI: 1.55–1.85]), and higher BMI (≥ 30 kg/m^2^, OR: 1.99 [95% CI: 1.64–2.41]) were independently associated with increased odds of FL (all *p* < 0.001). In contrast, Hispanic ethnicity (OR: 0.83 [95% CI: 0.74–0.92], *p* = 0.001), high income (OR: 0.50 [95% CI: 0.45–0.56], *p* < 0.001), private insurance (OR: 0.59 [95% CI: 0.54–0.65], *p* < 0.001), and full-time employment (OR: 0.43 [95% CI: 0.39–0.47], *p* < 0.001) were associated with lower odds of FL. Compared with married individuals, divorced/separated and widowed individuals had 21% (OR: 1.21 [95% CI: 1.12–1.31]) and 25% (OR: 1.25 [95% CI: 1.15–1.36]) higher odds of FL (both *p* < 0.001).

Among GI cancer survivors, there were consistent sex-based differences with women more likely to report any FLs (OR: 1.41 [95% CI 1.18–1.68]) but less likely to report any ALs (OR: 0.82 [95% CI 0.70–0.97]) (Supplementary Table 4 and Supplementary Table 5). In a subgroup analysis stratified by age, sex, and marital status, adjusted marginal prevalence of any AL was lowest among married females under 50 years (9.6% [95% CI: 9.2%−9.8%]) and highest among single males aged 50 years or older (24.9% [95% CI: 24.2%−25.7%]) when adjusted for covariates (Supplementary Table 6). As illustrated in Fig. 2a, across all age groups, divorced or widowed individuals had a higher AL prevalence compared with their married counterparts. Similar trends were seen for FL with the lowest adjusted prevalence among single males under 50 years (20.4% [95% CI: 19.9%−20.8%]) and highest among divorced or widowed females aged 50 years or older (52.0% [95% CI: 51.6%−52.5%]) (Supplementary Table 7). As demonstrated in Fig. 2b, the adjusted marginal FL prevalence increased with age, and individuals who were divorced or widowed consistently reported higher FL prevalence than their married and single counterparts.

## Discussion

Over the last several decades, major advancements in early detection and treatment have led to a growing population of long-term cancer survivors [30]. However, many survivors experience persistent functional impairments, highlighting gaps in post-treatment care [31]. Given the increasing prevalence of GI cancers, understanding long-term functional outcomes is important [32]. Specifically, many cancer survivors may experience persistent physical, cognitive, and functional impairments that affect their daily life [33]. Treatment-related complications (e.g., cachexia, bowel dysfunction, chronic pain, fatigue) can limit survivor mobility and daily activities [34, 35]. The current study was important as we defined cancer-related disabilities among GI cancer survivors compared with non-GI cancer survivors and the general US population. Using nationally representative NHIS data, we noted that nearly one-half of GI cancer survivors reported at least one AL—nearly four times the prevalence in the general US population and also higher than non-GI cancer survivors. Similarly, nearly three-quarters of GI cancer survivors reported FL, which was much more prevalent than non-GI cancer survivors and nearly twice that of the general population. Among different GI cancer types, pancreatic cancer survivors had the highest AL prevalence, whereas liver cancer survivors reported the greatest burden of FL. Importantly, several factors were associated with increased odds of AL and FL among GI survivors, including older age, higher BMI, lower income, unemployment, and being unmarried.

In recent years, there has been growing recognition that cancer survivorship extends beyond survival to include long-term functional disabilities that impact daily life. Prior research suggested that nearly 40% of cancer survivors experienced difficulties with mobility, cognition, and daily activities, yet these factors were infrequently recognized or addressed [36, 37]. As such, there is a need to incorporate patient-centered outcomes such as functional independence and quality of life into survivorship care and treatment decision-making [38]. While survival remains the dominant metric in oncology, other factors – including functional status – also is prioritized by patients [38]. For many patients, maintaining independence and daily functioning are valued as highly as, if not more than survival itself [39]. However, post-treatment disability is often overlooked when guiding complex treatment decisions [40, 41]. Findings in the current study serve to reinforce the importance of integrating QoL considerations into survivorship planning [42]. Cancer-related disabilities affect all aspects of daily living—from basic mobility to social participation and leisure activities—and can markedly compromise emotional well-being and independence [43, 44]. As patient preferences shift toward holistic, QoL-driven care, oncology practices should adapt to provide appropriate rehabilitation services, tailored survivorship care plans, and other interventions that preserve functional autonomy [45].

Social and demographic predictors can impact cancer survivor quality of life. For example, GI cancer survivors who were unmarried had greater functional limitations compared with their married counterparts. These data aligned with prior research noting that having a spouse or caregiver can facilitate post-treatment rehabilitation, encourage greater physical activity, and provide emotional support that improves recovery [46, 47]. Lack of such support among survivors may face higher disability [48, 49], with unmarried GI cancer survivors having a higher prevalence of disability than married survivors [50]. Notably, the protective effect of marriage was especially pronounced among men, consistent with evidence that men often derive greater survival and functional benefits from spousal support [51]. For example, Gomez et al. reported that unmarried men had a 27% higher risk of mortality than married men; in contrast, unmarried women had a 19% increased risk, suggesting that men may be more reliant on spousal caregiving to navigate post-cancer recovery [52]. In the current study, unmarried male GI cancer survivors more often had limitations in ADLS, IADLs, and routine tasks, reinforcing the importance of spousal caregiving to mitigate disability [53]. This disparity may stem from well-documented gender differences in health-seeking behaviors, with men being less likely than women to engage in proactive health management without encouragement from a partner [54].

Building on the broader impact of quality-of-life limitations, sociodemographic factors play a pivotal role in shaping functional outcomes among cancer survivors. Prior research has consistently highlighted disparities in disability prevalence based on income, insurance status, race, and social support networks [55]. Lower-income cancer survivors often experience higher risk of long-term physical and cognitive impairments due to reduced access to rehabilitative care and supportive services [56, 57]. GI cancer survivors in lower income groups and individuals with public insurance (e.g., Medicaid/Medicare) have reported higher rates of disability, likely reflecting limited access to supportive and rehabilitative care [58]. In the current study, unemployed cancer survivors had greater limitations versus individuals who were employed, which may have been related to both reduced resources and decreased physical activity or engagement in work-related health programs. These patterns highlight persistent disparities with survivors of lower socioeconomic status facing compounded barriers to recovery after treatment of a GI cancer [59]. Addressing these disparities will require equitable access to rehabilitation and support services across income and insurance groups [59].

Findings of the current study underscore the need for proactive, multidisciplinary survivorship care [60]. Clinicians should screen for and address functional impairments early in follow-up, connecting survivors with appropriate rehabilitation resources (e.g., physical therapy, occupational therapy, nutritional counseling) that facilitate independent living [60, 61]. Survivorship care plans should explicitly incorporate rehabilitation goals and encourage physical activity to maintain function [28, 62]. At a systems level, insurers and policymakers should ensure that cancer rehabilitation services are fully covered for all survivors independent of socioeconomic or employment status [63–65]. A structured, multidisciplinary care team — including oncologists, primary care providers, rehabilitation specialists (e.g., physical and occupational therapists, speech-language pathologists, dietitians) — is necessary to streamline transitions and provide comprehensive support [66, 67]. Future research should investigate the long-term trajectory of functional impairments and evaluate targeted interventions to improve quality of life and independence among GI cancer survivors [68].

The findings of the current study should be interpreted in the light of several limitations. The NHIS relies on self-reported data for cancer diagnoses and related limitations, which may be subject to recall and reporting biases. Whether reported functional impairments were directly attributable to cancer or its treatment versus other comorbidities could not be determined. The NHIS also lacked detailed clinical information (e.g., cancer stage, treatments, time since diagnosis), limiting our ability to assess how disease severity and specific therapies relate to functional outcomes. Additionally, gender-based reporting biases may have influenced the findings. For example, men might have underreported difficulties with daily activities while women may be more likely to report limitations. Despite these limitations, the current study provides important national insights into the functional outcomes of GI cancer survivors.

In conclusion, GI cancer survivors had a higher prevalence of cancer-related limitations than non-GI cancer survivors and the general population. These disparities were especially pronounced among survivors who were older, unmarried, of lower socioeconomic status, or reliant on public insurance. These results highlight the need for comprehensive survivorship care strategies that integrate early rehabilitation, tailored physical and psychosocial support, and policies to reduce disparities in post-treatment recovery. Addressing these needs will help preserve independence and quality of life for GI cancer survivors.

## Supplementary Information

Below is the link to the electronic supplementary material.

Supplementary file1 (DOCX 51 KB)

Supplementary file2 Prevalence of specific cancer-related limitations among gastrointestinal (GI) cancer survivors, non-GI cancer survivors, and the general U.S. population. Weighted percentages and standard errors shown. (PNG 83 KB)

## Data Availability

The data for this study were obtained from the Integrated Public Use Microdata Series National Health Interview Survey (IPUMS NHIS), which is publicly available. The datasets analyzed during the current study can be accessed through the IPUMS NHIS repository at https://nhis.ipums.org.
