# Supplementary material for: Prevalence and trends of cancer-related daily life limitations among gastrointestinal cancer survivors
Source: J Cancer Surviv. 2025 May 24;20(Suppl 1):375–84. doi: 10.1007/s11764-025-01833-1 (PMC13676644; doi:10.1007/s11764-025-01833-1)
Supplement: Supplementary file 1 [file 11764_2025_1833_MOESM1_ESM.docx]

**Supplementary Table 1.** Cancer Type Distribution Among Non-Gastrointestinal Cancer Survivors in the Study Cohort.

| Characteristic | Non-GI Cancer Survivors  (n = 39,887) |
| --- | --- |
| Breast cancer | 12,982 (31.7%) |
| Prostate cancer | 8,195 (19.6%) |
| Lung cancer | 2,183 (5.2%) |
| Kidney cancer | 1,056 (3.5%) |
| Bladder cancer | 1,856 (4.5%) |
| Thyroid cancer | 1,741 (4.0%) |
| Cervical cancer | 4,707 (12.5%) |
| Ovarian cancer | 1,732 (4.2%) |
| Testicular cancer | 413 (1.4%) |
| Uterine cancer | 3,473 (9.2%) |
| Head and neck cancer | 1,281 (3.2%) |
| Soft tissue cancer | 268 (0.9%) |

^*N represents the unweighted number of survey respondents. Weighted percentages are reported.^

**Supplementary Table 2.** Multivariable logistic regression analysis of factors associated with any activity limitation (AL) along with adjusted prevalence among GI and non-GI cancer survivors.

| Variables | Adjusted prevalence of AL (95% CI) | Multivariable logistic regression | |
| --- | --- | --- | --- |
|  |  | Adjusted OR (95% CI) | *P* value |
| Cancer type |  |  |  |
| Non-GI cancer | 35.9% (35.2-36.7) | Reference |  |
| GI cancer | 40.5% (38.6-42.4) | 1.21 (1.11-1.32) | <0.001 |
| Sex |  |  |  |
| Male | 38.5% (37.3-39.6) | Reference |  |
| Female | 35.4% (34.5-36.2) | 0.88 (0.82-0.93) | <0.001 |
| Age (years) |  |  |  |
| <50 | 30.6% (28.6-32.6) | Reference |  |
| ≥50 | 37.6% (36.8-38.4) | 1.36 (1.23-1.52) | <0.001 |
| BMI (kg/m^2^) |  |  |  |
| <18.5 | 49.5% (45.1-53.9) | Reference |  |
| 18.5-24.9 | 32.7% (31.6-33.9) | 0.50 (0.41-0.60) | <0.001 |
| 25-29.9 | 32.1% (31.1-33.2) | 0.48 (0.40-0.58) | <0.001 |
| ≥30 | 45.1% (43.9-46.4) | 0.84 (0.70-1.00) | 0.059 |
| Race |  |  |  |
| White | 36.3% (35.5-37.0) | Reference |  |
| Black | 36.6% (34.7-38.6) | 1.02 (0.93-1.11) | 0.740 |
| Other | 39.3% (36.3-42.3) | 1.14 (0.99-1.29) | 0.056 |
| Ethnicity |  |  |  |
| Non-Hispanic | 36.8% (36.0-37.5) | Reference |  |
| Hispanic | 33.3% (31.0-35.7) | 0.86 (0.77-0.96) | 0.009 |
| Citizenship |  |  |  |
| Citizen | 36.6% (35.9-37.3) | Reference |  |
| Immigrant | 30.0% (25.7-34.3) | 0.74 (0.60-0.91) | 0.004 |
| Income |  |  |  |
| Below poverty line | 54.2% (52.2-56.3) | Reference |  |
| Low to middle income | 44.8% (43.3-46.2) | 0.68 (0.62-0.75) | <0.001 |
| High income | 30.2% (29.4-31.0) | 0.37 (0.33-0.40) | <0.001 |
| Insurance type |  |  |  |
| Medicare | 38.0% (37.0-39.0) | Reference |  |
| Medicaid | 48.1% (44.7-51.5) | 1.51 (1.30-1.74) | <0.001 |
| Other public | 37.7% (32.5-43.0) | 0.99 (0.78-1.24) | 0.913 |
| Private | 31.8% (30.3-33.4) | 0.76 (0.69-0.83) | <0.001 |
| Uninsured | 32.5% (29.6-35.4) | 0.78 (0.68-0.91) | 0.001 |
| Marital status |  |  |  |
| Married | 31.6% (30.6-32.6) | Reference |  |
| Single | 39.1% (36.8-41.5) | 1.39 (1.25-1.55) | <0.001 |
| Divorced/separated | 43.1% (41.6-44.6) | 1.64 (1.52-1.77) | <0.001 |
| Widowed | 39.4% (37.9-40.8) | 1.40 (1.30-1.51) | <0.001 |
| Employment status |  |  |  |
| Unemployed | 46.8% (46.0-47.7) | Reference |  |
| Part time | 24.7% (22.9-26.5) | 0.37 (0.34-0.41) | <0.001 |
| Full time | 16.5% (15.3-17.8) | 0.22 (0.20-0.25) | <0.001 |

_Abbreviations: AL: activity limitation; BMI: Body mass index; CI: Confidence interval; FL: Functional limitation; GI: Gastrointestinal; OR: Odds ratio_

**Supplementary Table 3.** Multivariable logistic regression analysis of factors associated with any functional limitations (FL) along with adjusted prevalence among GI and non-GI cancer survivors.

| Variables | Adjusted prevalence of FL | Multivariable logistic regression | |
| --- | --- | --- | --- |
|  |  | Adjusted OR (95% CI) | *P* value |
| Cancer type |  |  |  |
| Non-GI cancer | 68.1% (67.5-68.8) | Reference |  |
| GI cancer | 69.9% (68.1-71.7) | 1.11 (1.00-1.19) | 0.049 |
| Sex |  |  |  |
| Male | 63.6% (62.4–64.7) | Reference |  |
| Female | 70.9% (70.1–71.7) | 1.40 (1.31-1.49) | <0.001 |
| Age (years) |  |  |  |
| <50 | 58.1% (56.1–60.0) | Reference |  |
| ≥50 | 70.1% (69.5–70.8) | 1.69 (1.55-1.85) | <0.001 |
| BMI (kg/m^2^) |  |  |  |
| <18.5 | 66.4% (62.3–70.5) | Reference |  |
| 18.5-24.9 | 58.7% (57.6–59.9) | 0.72 (0.59-0.87) | 0.001 |
| 25-29.9 | 65.7% (64.7–66.8) | 0.97 (0.80-1.17) | 0.759 |
| ≥30 | 79.7% (78.8–80.7) | 1.99 (1.64-2.41) | <0.001 |
| Race |  |  |  |
| White | 68.8% (68.1–69.5) | Reference |  |
| Black | 64.9% (62.9–66.9) | 0.84 (0.76-0.92) | <0.001 |
| Other | 68.0% (65.2–70.8) | 0.96 (0.85-1.09) | 0.574 |
| Ethnicity |  |  |  |
| Non-Hispanic | 68.7% (68.0–69.3) | Reference |  |
| Hispanic | 64.4% (62.0–66.9) | 0.83 (0.74-0.92) | 0.001 |
| Citizenship |  |  |  |
| Citizen | 68.4% (67.8–69.1) | Reference |  |
| Immigrant | 65.4% (61.0–69.8) | 0.87 (0.72-1.06) | 0.174 |
| Income |  |  |  |
| Below poverty line | 77.8% (76.2–79.5) | Reference |  |
| Low to middle income | 74.3% (73.1–75.6) | 0.83 (0.74-0.92) | 0.001 |
| High income | 63.6% (62.8–64.5) | 0.50 (0.45-0.56) | <0.001 |
| Insurance type |  |  |  |
| Medicare | 71.8% (70.9–72.6) | Reference |  |
| Medicaid | 73.1% (70.1–76.0) | 1.07 (0.91-1.25) | 0.413 |
| Other public | 67.1% (62.3–72.0) | 0.80 (0.64-1.01) | 0.061 |
| Private | 60.1% (58.6–61.6) | 0.59 (0.54-0.65) | <0.001 |
| Uninsured | 63.9% (61.0–66.8) | 0.70 (0.61-0.80) | <0.001 |
| Marital status |  |  |  |
| Married | 66.3% (65.3–67.2) | Reference |  |
| Single | 66.8% (64.6–68.9) | 1.02 (0.92-1.14) | 0.678 |
| Divorced/separated | 70.4% (69.0–71.7) | 1.21 (1.12-1.31) | <0.001 |
| Widowed | 71.1% (69.7–72.4) | 1.25 (1.15-1.36) | <0.001 |
| Employment status |  |  |  |
| Unemployed | 73.5% (72.7–74.2) | Reference |  |
| Part time | 59.8% (57.8–61.8) | 0.54 (0.49-0.59) | <0.001 |
| Full time | 54.4% (52.6-56.1) | 0.43 (0.39-0.47) | <0.001 |

_Abbreviations: BMI: Body mass index; CI: Confidence interval; FL: Functional limitation; GI: Gastrointestinal; OR: Odds ratio_

**Supplementary Table 4.** Multivariable logistic regression analysis of factors associated with any activity limitation (AL) along with adjusted prevalence, stratified by GI cancer survivors.

| Variables | Adjusted prevalence of AL | Multivariable logistic regression | |
| --- | --- | --- | --- |
|  |  | Adjusted OR (95% CI) | *P* value |
| Sex |  |  |  |
| Male | 48.5% (45.7–51.4) | Reference |  |
| Female | 43.7% (40.8–46.5) | 0.82 (0.70-0.97) | 0.022 |
| Age (years) |  |  |  |
| <50 | 53.7% (45.6–61.8) | Reference |  |
| ≥50 | 45.4% (43.3–47.5) | 0.72 (0.51–1.02) | 0.061 |
| BMI (kg/m^2^) |  |  |  |
| <18.5 | 63.4% (53.7–73.0) | Reference |  |
| 18.5-24.9 | 44.8% (41.4–48.1) | 0.47 (0.30–0.73) | 0.001 |
| 25-29.9 | 41.6% (38.3–44.9) | 0.41 (0.27-0.64) | <0.001 |
| ≥30 | 50.8% (47.1–54.5) | 0.59 (0.38-0.93) | 0.022 |
| Race |  |  |  |
| White | 46.0% (43.8–48.2) | Reference |  |
| Black | 46.3% (40.6–51.9) | 1.00 (0.79-1.29) | 0.943 |
| Other | 47.0% (39.1–54.9) | 1.04 (0.75-1.45) | 0.816 |
| Ethnicity |  |  |  |
| Non-Hispanic | 68.7% (68.0–69.3) | Reference |  |
| Hispanic | 64.4% (62.0–66.9) | 0.91 (0.67-1.23) | 0.537 |
| Citizenship |  |  |  |
| Citizen | 46.2% (44.2–48.2) | Reference |  |
| Immigrant | 43.4% (30.7–56.1) | 0.89 (0.53-1.51) | 0.676 |
| Marital status |  |  |  |
| Married | 40.8% (37.7–43.9) | Reference |  |
| Single | 52.5% (45.5–59.4) | 1.60 (1.18-2.18) | 0.003 |
| Divorced/separated | 52.7% (48.1–57.2) | 1.61 (1.29-2.02) | <0.001 |
| Widowed | 47.8% (44.0–51.6) | 1.33 (1.09-1.63) | 0.006 |
| Income |  |  |  |
| Below poverty line | 58.4% (53.2–63.7) | Reference |  |
| Low to middle income | 53.4% (49.6–57.3) | 0.82 (0.63-1.06) | 0.126 |
| High income | 39.7% (37.2–42.3) | 0.47 (0.37-0.60) | <0.001 |
| Insurance type |  |  |  |
| Medicare | 46.5% (43.9–49.0) | Reference |  |
| Medicaid | 62.5% (52.6–72.4) | 1.92 (1.24-2.98) | 0.004 |
| Other public | 64.1% (45.6–82.6) | 2.06 (0.92-4.64) | 0.082 |
| Private | 40.8% (35.4–46.2) | 0.79 (0.61-1.04) | 0.090 |
| Uninsured | 41.1% (30.4–51.8) | 0.80 (0.51-1.28) | 0.359 |
| Employment status |  |  |  |
| Unemployed | 54.2% (51.9–56.5) | Reference |  |
| Part time | 36.6% (30.1–43.2) | 0.49 (0.36-0.66) | <0.001 |
| Full time | 18.4% (14.0–22.7) | 0.19 (0.14-0.26) | <0.001 |

_Abbreviations: AL: activity limitation; BMI: Body mass index; CI: Confidence interval; GI: Gastrointestinal; OR: Odds ratio_

**Supplementary Table 5.** Multivariable logistic regression analysis of factors associated with any functional limitation (FL) along with adjusted prevalence, stratified by GI cancer survivors.

| Variables | Adjusted prevalence of FL | Multivariable logistic regression | |
| --- | --- | --- | --- |
|  |  | Adjusted OR (95% CI) | *P* value |
| Sex |  |  |  |
| Male | 68.4% (65.8-70.9) | Reference |  |
| Female | 75.3% (72.9-77.7) | 1.41 (1.18-1.68) | <0.001 |
| Age (years) |  |  |  |
| <50 | 71.0% (64.8-77.2) | Reference |  |
| ≥50 | 72.0% (70.2-73.8) | 1.05 (0.76-1.44) | 0.771 |
| BMI (kg/m^2^) |  |  |  |
| <18.5 | 78.0% (69.8-86.3) | Reference |  |
| 18.5-24.9 | 65.4% (62.2-68.5) | 0.53 (0.32-0.88) | 0.013 |
| 25-29.9 | 69.1% (66.2-72.2) | 0.63 (0.38-1.04) | 0.073 |
| ≥30 | 80.2% (77.5-83.0) | 1.14 (0.68-1.90) | 0.612 |
| Race |  |  |  |
| White | 72.3% (70.4-74.2) | Reference |  |
| Black | 68.6% (63.4-73.9) | 0.84 (0.64-1.09) | 0.190 |
| Other | 72.8% (65.9-79.6) | 1.02 (0.72-1.47) | 0.893 |
| Ethnicity |  |  |  |
| Non-Hispanic | 72.3% (70.5-74.1) | Reference |  |
| Hispanic | 67.0% (60.5-73.6) | 0.78 (0.57-1.06) | 0.115 |
| Citizenship |  |  |  |
| Native | 71.9% (70.1-73.65) | Reference |  |
| Immigrant | 71.7% (60.5-82.8) | 0.99 (0.57-1.73) | 0.970 |
| Marital status |  |  |  |
| Married | 71.0% (68.3-73.7) | Reference |  |
| Single | 70.8% (64.8-76.9) | 0.99 (0.72-1.37) | 0.964 |
| Divorced/separated | 73.8% (69.9-77.6) | 1.15 (0.91-1.45) | 0.252 |
| Widowed | 72.2% (68.8-76.9) | 1.06 (1.85-1.33) | 0.590 |
| Income |  |  |  |
| Below poverty line | 80.4% (76.3-84.6) | Reference |  |
| Low to middle income | 75.8% (72.5-79.1) | 0.76 (0.56-1.05) | 0.095 |
| High income | 67.3% (64.9-69.7) | 0.50 (0.37-0.68) | <0.001 |
| Insurance type |  |  |  |
| Medicare | 73.8% (71.7-75.9) | Reference |  |
| Medicaid | 74.9% (66.4-83.5) | 1.06 (0.66-1.71) | 0.808 |
| Other public | 83.4% (70.4-96.3) | 1.78 (0.69-4.55) | 0.232 |
| Private | 64.2% (59.3-69.1) | 0.64 (0.49-0.82) | <0.001 |
| Uninsured | 64.4% (54.2-74.5) | 0.64 (0.40-1.02) | 0.062 |
| Employment status |  |  |  |
| Unemployed | 76.0% (74.1-77.9) | Reference |  |
| Part time | 61.4% (54.9-67.8) | 0.50 (0.37-0.67) | <0.001 |
| Full time | 53.9% (48.1-59.7) | 0.37 (0.28-0.48) | <0.001 |

_Abbreviations: BMI: Body mass index; CI: Confidence interval; FL: Functional limitation; GI: Gastrointestinal; OR: Odds ratio_

**Supplementary Table 6.** Stratified analysis of adjusted prevalence between sex, age, and marital status interactions among GI and non-GI cancer survivors with any activity limitation (AL).

| Variables | Adjusted Prevalence of AL (95% CI) |
| --- | --- |
|  |  |
| Sex x Marital status x Age |  |
| Female x Married x <50 yrs | 9.6% (9.2%-9.8%) |
| Male x Married x <50 yrs | 12.6% (12.2%-12.9%) |
| Female x Single x <50 yrs | 9.5% (9.2%-9.8%) |
| Male x Single x <50 yrs | 13.3% (12.9%-13.7%) |
| Female x Other x <50 yrs | 17.0% (16.5%-17.5%) |
| Male x Other x <50 yrs | 22.3% (21.6%-23.1%) |
| Female x Married x ≥50 yrs | 16.8% (16.5%-17.1%) |
| Male x Married x ≥50 yrs | 17.9% (17.6%-18.2%) |
| Female x Single x ≥50 yrs | 23.1% (22.4%-23.9%) |
| Male x Single x ≥50 yrs | 24.9% (24.2%-25.7%) |
| Female x Other x ≥50 yrs | 23.0% (22.7%-23.2%) |
| Male x Other x ≥50 yrs | 24.6% (24.2%-24.9%) |

_All p-values are <0.001. *Divorced/Separated/Widowed_

**Supplementary Table 7.** Stratified analysis of adjusted prevalence between sex, age, and marital status interactions among GI and non-GI cancer survivors with any functional limitation (FL).

| Variables | Adjusted Prevalence of FL (95% CI) |
| --- | --- |
|  |  |
| Sex x Marital status x Age |  |
| Female x Married x <50 yrs | 29.3% (28.9%-29.6%) |
| Male x Married x <50 yrs | 25.9% (25.5%-26.3%) |
| Female x Single x <50 yrs | 24.7% (24.2%-25.1%) |
| Male x Single x <50 yrs | 20.4% (19.9%-20.8%) |
| Female x Other x <50 yrs | 35.5% (34.9%-36.1%) |
| Male x Other x <50 yrs | 31.2% (30.5%-32.0%) |
| Female x Married x ≥50 yrs | 46.2% (45.8%-46.6%) |
| Male x Married x ≥50 yrs | 38.6% (38.2%-39.0%) |
| Female x Single x ≥50 yrs | 50.3% (49.3%-51.3%) |
| Male x Single x ≥50 yrs | 38.8% (37.9%-39.8%) |
| Female x Other x ≥50 yrs | 52.0% (51.6%-52.5%) |
| Male x Other x ≥50 yrs | 42.1% (41.5%-42.7%) |

_All p-values are <0.001. *Other: Divorced/Separated/Widowed_
